# A Retrospective Comparative Cohort Study of Craniotomy and Prophylactic Enoxaparin Timing

**DOI:** 10.7759/cureus.23867

**Published:** 2022-04-06

**Authors:** David R Hallan, Bao Sciscent, Elias Rizk

**Affiliations:** 1 Neurosurgery, Penn State Health Milton S. Hershey Medical Center, Hershey, USA

**Keywords:** outcomes, enoxaparin, lovenox, mortality rate, myocardial infarction, pulmonary embolism, dvt, prophylaxis, craniotomy, neurosurgery

## Abstract

Introduction: Post-operative venous thromboembolism (VTE) prophylaxis is the standard of care after craniotomy, but there is debate over when to initiate VTE prophylaxis to decrease the morbidity and mortality experienced by these patients. This study aims to determine the effects of starting enoxaparin on day one vs. day three after craniotomy.

Methods: We used a multi-institutional health research network (TriNetX) to gather data from the electronic medical records of patients who started enoxaparin one day after craniotomy (cohort 1) and patients who started it three days later (cohort 2). Our primary endpoint was mortality, with the secondary endpoints of deep venous thrombosis (DVT), additional craniotomy, pulmonary embolism (PE), myocardial infarction (MI), ischemic stroke (IS), intracerebral hemorrhage (ICH), ventilator and tracheostomy dependence, or percutaneous endoscopic gastrostomy (PEG) tube dependence. Patients were propensity score-matched for demographics, common comorbidities, and anticoagulant and antiplatelet use.

Results: After propensity score matching, 1,554 patients were identified in each cohort. In cohort 1, 21.171% of patients were deceased after five years vs. 26.126% in cohort 2 (p= 0.0012; OR 0.759, 95% CI (0.643,0.897)). The 30-day survival was 94.521% vs. 93.049%, the 90-day survival was 90.200% vs. 87.335%, and the 365-day survival was 80.619 vs. 76.817%. Deep venous thrombosis occurred in 5.277% of cohort 1 and 7.851% of cohort 2 (p=0.0038, OR 0.654, 95% CI [0.49,0.873]). There was no increase in intracerebral hemorrhage in cohort 1. There were no statistically significant differences in subsequent craniotomy rates, PE, MI, IS, ventilator/tracheostomy, or PEG tube dependence.

Conclusion: Starting enoxaparin on day one after craniotomy was associated with decreased mortality and DVTs, with no difference in rates of PE, MI, IS, tracheostomy/PEG dependence, or further craniotomy.

## Introduction

Venous thromboembolism (VTE) is a common and potentially fatal postoperative complication of neurological surgery. The development of VTE is multifactorial and depends on the patient's age, comorbidities, and type of surgery performed [1]. Some incidences of VTE have been reported to be greater than 20%, especially in patients with glioblastoma. This supports the use of pharmacologic prophylaxis after craniotomy despite the serious risk of intracranial hemorrhage (ICH) [2-5]. Low molecular weight heparins, such as enoxaparin, have been shown to reduce the risk of developing lower extremity deep venous thrombosis (DVT) without severe risk of ICH [6]. However, there is debate over when to initiate VTE pharmacologic prophylaxis after brain surgery [7-8]. Appropriate timing of prophylactic treatment is crucial to prevention of long-term complications and increased mortality.

We sought to determine the impact of starting enoxaparin on day one after craniotomy vs. day three, with the primary endpoints of mortality, intracerebral hemorrhage, DVT, pulmonary embolism (PE), ischemic stroke (IS), myocardial infarction (MI), and secondary endpoints of ventilator/tracheostomy dependence and percutaneous endoscopic gastrostomy (PEG tube) dependence.

## Materials and methods

This was a retrospective comparative cohort study. We used a multi-institutional healthcare database, the TriNetX research network, to collate information on patients who underwent craniotomy and subsequently were started on prophylactic enoxaparin one day after (cohort 1) vs. three days after (cohort 2) craniotomy. The TriNetX research network provides access to 56 health care organizations (HCOs) and their de-identified electronic medical record data. Data includes demographics, diagnoses, medications, laboratory values, genomics, and procedures. The identity of the HCOs and patients is not disclosed to comply with ethical guidelines against data re-identification. Due to the database's federated nature, an institutional review board (IRB) waiver was granted. The data is updated daily. Our use of this database and its validity were informed by previous literature, and the exact details of the network have been previously described [9-12].

Data extraction and analysis were performed on September 9, 2021. Datasets were retrospectively queried using diagnostic International Classification of Diseases, tenth revision, clinical modification (ICD-10) and current procedural terminology codes. The medical information included age at index date, sex, race, and the comorbidities of hypertension, acute kidney injury, diabetes, ischemic heart disease, heart failure, atrial fibrillation, disorders of lipoprotein metabolism and other lipidemias, obesity, history of nicotine dependence, chronic respiratory disease, cirrhosis, alcohol abuse or dependence, and peripheral vascular disease. These were recorded up to the date of the index date, which was set on the day of craniotomy. 

The Analysis was performed using unmatched and propensity score-matched cohorts, using the greedy-nearest neighbor algorithm with a calliper of 0.1 pooled standard deviations. Hazard ratios were calculated using R's survival package v3.2-3 and were validated by comparing the output to SAS version 9.4. (SAS Institute Inc., Cary, NC, USA) Chi-square analysis that was performed on categorical variables. Our primary outcome of interest was mortality and DVT, with secondary outcomes of PE, IS, MI, tracheostomy and PEG. Outcomes were examined over five years, with interval analyses at 30-, 90-, and 365-days. Statistical significance was set at p<0.05.

## Results

After propensity score matching, 1,154 patients were identified in each cohort. The mean age at craniotomy of cohort 1 was 54.6 ± 17.4 years and 55.5 ± 17.9 years for cohort 2. Patients of the male sex comprised 53% of cohort 1 and 57% of cohort 2. Around 76% of cohort 1 and 81% of cohort 2 were Caucasian. Baseline demographics can be seen in Table 1.

**Table 1:** Baseline demographics and characteristics after propensity score matching ICD = International Classification of Diseases, n = number, Std = Standard

ICD Code	Diagnosis	Cohort 1 n	n % of Cohort 1	Cohort 2 n	n % of Cohort2	p-Value	Std Mean Difference
AI	Age at Index	1,554	100%	1,554	100%	0.7014198	0.013755836
2106-3	Caucasian	1,190	76.58%	1,208	77.74%	0.4418587	0.027592437
M	Male	823	52.96%	806	51.87%	0.5414756	0.021905862
F	Female	731	47.04%	747	48.07%	0.5655047	0.020617787
2054-5	African American	188	12.10%	183	11.78%	0.78207064	0.009923868
2131-1	Unknown race	152	9.78%	141	9.07%	0.49952132	0.02422599
2028-9	Asian	15	0.97%	11	0.71%	0.4308333	0.028263787
I10-I16	Hypertensive diseases	837	53.86%	806	51.87%	0.26530185	0.039970614
E78	Disorders of lipoprotein metabolism and other lipidemias	488	31.40%	478	30.76%	0.6983395	0.013904078
R53	Malaise and fatigue	369	23.75%	356	22.91%	0.5813716	0.019781698
Z87.891	Personal history of nicotine dependence	364	23.42%	358	23.04%	0.7988375	0.009142858
F17	Nicotine dependence	354	22.78%	339	21.82%	0.5180153	0.023191309
R13	Aphagia and dysphagia	319	20.53%	291	18.73%	0.20603123	0.0453773
E08-E13	Diabetes mellitus	306	19.69%	304	19.56%	0.9280287	0.003240406
R40	Somnolence, stupor and coma	289	18.60%	265	17.05%	0.2606616	0.040361222
E65-E68	Overweight, obesity and other hyperalimentation	288	18.53%	278	17.89%	0.6420913	0.016674357
J40-J47	Chronic lower respiratory diseases	269	17.31%	255	16.41%	0.50238377	0.02406454
I20-I25	Ischemic heart diseases	209	13.45%	207	13.32%	0.9160881	0.003779867
N17-N19	Acute kidney failure and chronic kidney disease	180	11.58%	187	12.03%	0.69720894	0.013958896
I48	Atrial fibrillation and flutter	159	10.23%	154	9.91%	0.7656868	0.01069161
R63	Symptoms and signs concerning food and fluid intake	141	9.07%	144	9.27%	0.85208464	0.006689225
I50	Heart failure	92	5.92%	89	5.73%	0.8182632	0.008243361
F10.1	Alcohol abuse	70	4.51%	75	4.83%	0.67064273	0.015256777
I73	Other peripheral vascular diseases	57	3.67%	53	3.41%	0.6977802	0.013931195
F10.2	Alcohol dependence	51	3.28%	45	2.90%	0.5339058	0.022317482
K74	Fibrosis and cirrhosis of the liver	12	0.77%	14	0.90%	0.69366723	0.014130835
1191	Aspirin	543	34.94%	558	35.91%	0.57373846	0.02018255
11289	Warfarin	131	8.43%	131	8.43%	1	0
8410	Alteplase	105	6.76%	117	7.53%	0.40327314	0.029987164
1364430	Apixaban	59	3.80%	61	3.93%	0.8522846	0.006680077
1114195	Rivaroxaban	36	2.32%	42	2.70%	0.49141815	0.024685714
259280	Tenecteplase	10	0.64%	10	0.64%	1	0
31500	Intubation, endotracheal, emergency procedure	116	7.47%	119	7.66%	0.83870924	0.007302178

**Figure 1:**
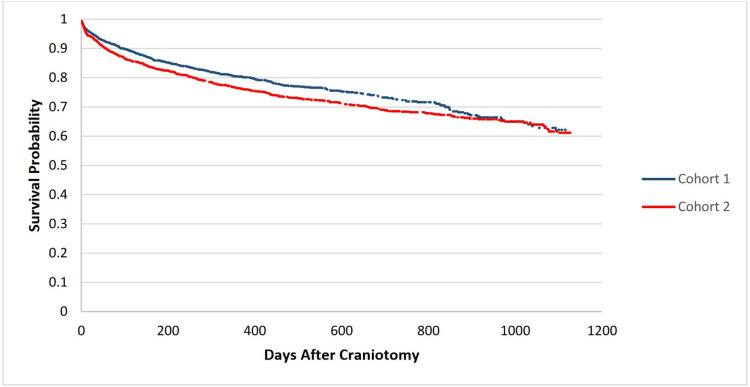
Kaplan-Meier survival curve for the outcome of deceased comparing cohort 1 vs. cohort 2

Table 2 shows measures of association between the day of enoxaparin use after craniotomy and mortality. After propensity score matching, 329 (21.171%) of patients in the day-one cohort died vs. 406 (26.126%) in the day-three cohort. The risk difference was -4.955% (95% CI -7.938, -1.972%); risk ratio (RR) 0.81, 95% confidence interval (CI) (0.714,0.92); odds ratio (OR) 0.759, 95% CI (0.643,0.897; p=0.0012). Figure 1 shows a Kaplan-Meier survival curve for the outcome of deceased comparing cohort 1 vs. cohort 2. The 30-day survival rate was 94.5% vs. 93.0% respectively, with a 90-day survival of 90.2% vs. 87.3%, 365-day survival of 80.6% vs. 76.8% and a five-year survival probability of 62.1% vs. 61.1% (p=0.0542, hazard ratio 0.867, 95% CI [0.749, 1.003]).

**Table 2:** Measures of association between the day of enoxaparin use after craniotomy and mortality

Cohort	Patients in cohort	Patients deceased	Risk	Risk difference	Risk ratio	Odds ratio
1	1,554	329	21.171%	-4.955% (-7.938,-1.972%)	0.81 (0.714,0.92)	0.859 (0.643,0.897)
2	1,554	406	26.126%	p=0.0012		

Table 3 shows outcomes after propensity score matching. Deep vein thrombosis occurred in 5.28% of cohort 1 and 7.85% of cohort 2 (p=0.0038, OR 0.654, 95% CI [0.49,0.873]). The DVT rates at 30 days were 2.219% vs. 3.329% (p=0.0543) and 3.206% vs. 4.932% (p=0.0128) at 90 days. There were comparable rates of respirator (4.44% vs. 4.25%, OR 1.048 [0.742-1.479]), dependence tracheostomy (4.89% vs. 6.24%, OR 0.772 [0.567-1.052]), PEG (5.15% vs. 6.05%, OR 0.843 [0.62-1.146]), subsequent craniotomy (7.66% vs. 8.88%, OR 0.851 [0.659-1.099]), PE (5.79% vs. 7.14%, OR 0.799 [0.6-1.065], stroke (3.86% vs. 3.28%, OR 1.184 [0.809, 1.731]), TIA (0.90% vs. 1.42%, OR 0.633 [0.323, 1.242]), and MI (2.25% vs. 2.25%, OR [0.623, 1.606]) between cohorts.

**Table 3:** Risks of each outcome measure after administration of enoxaparin day one (cohort 1) vs. day three (cohort 2) post-craniotomy

Outcome	Cohort 1	Cohort 2	Significant values
Deceased	329 (21.171%)	406 (26.126%)	p= 0.0012 OR 0.750 (0.643, 0.897)
Dependence on respirator	69 (4.44%)	69 (4.247%)	p= 0.7918 OR 1.048 (0.742, 1.479)
Tracheostomy	76 (4.891%)	97 (6.242%)	p=0.1004 OR 0.772 (0.567, 1.052)
Gastrostomy tube	80 (5.148%)	94 (6.0495%)	p=0.2747 OR 0.843 (0.62, 1.146)
Craniotomy	119 (7.658%)	138 (8.88%)	p=0.2159 OR 0.851 (0.659, 1.099)
Pulmonary embolism	90 (5.792%)	111 (7.143%)	p=0.1256 OR 0.799 (0.6, 1.065)
Deep venous thrombosis	82 (5.277%)	122 (7.851%)	p=0.0038 OR 0.654 (0.49,0.873)
Intracranial hemorrhage	128 (8.237%)	167 (10.746%)	p=0.0170 OR 0.746 (0.585, 0.949)
Ischemic stroke	60 (3.861%)	51 (3.282%)	p=0.3843 OR 1.184 (0.809, 1.731)
Transient ischemic attack	14 (0.901%)	22 (1.416%)	p=0.1799 OR 0.633 (0.323, 1.242)
Myocardial infarction	35 (2.252%)	35 (2.252%)	p=1 OR 1 (0.623, 1.606)

## Discussion

In this study, we present outcomes of patients who were administered prophylactic enoxaparin following craniotomy. Based on our analysis, the initiation of enoxaparin within 24 hours after surgery was associated with improved mortality and DVT outcomes.

Current guidelines recommend use of prophylactic anticoagulation in neurosurgical patients following craniotomy. However, individual factors in deciding the timing of anticoagulation vary. For example, brain malignancies such as glioblastoma multiforme, which cause a pro-thrombotic state, put patients at an increased risk of VTE [13-14]. Risk factors for postoperative ICH include older age, larger tumor size, and increased operative time among others [15-17].

Previous studies report a range of rates of VTE and ICH following craniotomy after administration or prophylactic anticoagulation. Briggs et al. analyzed 1,087 patients who underwent craniotomy for tumor resection. Their study observed increased rates of VTE in patients with a high-grade glioma and increased operative time. However, prophylactic enoxaparin initiation within 72 hours decreased likelihood of VTE. Furthermore, rates of ICH were comparable between those who did and did not receive enoxaparin [8]. A similar report by Algattas et al. compared adult patients who received anticoagulation prophylaxis after undergoing craniotomy for tumor on day one and day two after surgery and reported no significant differences in risk of VTE or ICH. Conversely, a retrospective study by Cage et al. concluded that enoxaparin use within 48 hours after resections of meningiomas are associated with lower rate of VTE and noted no differences in the incidence of intracranial hemorrhage in these patients [18]. Other studies looked into VTE prophylaxis initiation at time of surgery. However, reports differ on its safety and the risk of ICH [19-20].

As shown in our study and prior reports, there is a narrow window for the optimal use of prophylactic enoxaparin. A delicate balance between individual potential risks and benefits must be considered. Timing of appropriate anticoagulation prophylaxis is multifactorial and complications of VTE and ICH can significantly decrease quality of life.

Our analysis was not without limitations. In our analysis, the exact neuropathology that required the craniotomy, the specific type of craniotomy technique used, and the total operative time were not identified. Also, the frequency in screening for VTE may account for some variation in results from our study and those previously reported. A disproportionate majority of our study population was Caucasian and so our results may not be generalized to all racial groups. The major limitation of this study was that it was retrospective in nature. Furthermore, due to the nature of the database, we were unable to collect patient level data on specific outcomes. We were unable to report on imaging information. We do not have information on the type of diagnostic test used for confirmation of disease. The data collected was for billing purposes, not for clinical use, and thus much clinical information is missing. In addition, some misidentification is inevitable in database studies.

## Conclusions

Initiation of VTE prophylaxis on day one after craniotomy may provide a survival benefit for patients compared to administration on day three, as well as decrease the risk of DVTs. Studies that provide patient level data such as the neuropathology that required the initial craniotomy as well as the screening frequency for outcomes such as DVT and MI may offer additional insight into the optimal timing of anticoagulation prophylaxis.
